# Digital transformation and enterprise resilience: Enabling or burdening?

**DOI:** 10.1371/journal.pone.0305615

**Published:** 2024-07-19

**Authors:** Cheng Li, Yewen Wang

**Affiliations:** School of Economics and Management, Tiangong University, Tianjin, China; Zhejiang University of Technology, CHINA

## Abstract

Based on data from A-share listed companies from 2007 to 2021, this study constructs an enterprise resilience index using the entropy-weighted TOPSIS method. On this basis, the impact of digital transformation on enterprise resilience is examined through empowerment theory. The empirical results indicate that digital transformation significantly enhances enterprise resilience, a conclusion that remains valid after a series of robustness tests. Mechanism analysis reveals that digital transformation enhances enterprise resilience by reducing agency costs, increasing information transparency, and alleviating financing constraints. Heterogeneity analysis shows that micro-characteristics of enterprises, such as the stage of the life cycle and factor intensity, as well as macro-environmental factors like regional intellectual property rights protection and industry competition level, have differential impacts on the resilience-enhancing effects of digital transformation. Further analysis suggests that the level of corporate governance influences the positive impact of digital transformation on enterprise resilience.

## 1. Introduction

Since the global financial crisis 2008, major risk events have occurred frequently, leading to complex and severe global economic conditions. The COVID-19 pandemic in 2020 has further had a profound impact on the global economic and political structure. As economic, political, and sudden public health events intertwine, businesses face significant survival challenges. Organizational adaptability theory suggests that gaps in expectations or declines in organizational performance can trigger strategic transformations. In the era of the digital economy, characterized by rapid technological advancements and the emergence of new business models and patterns, digital transformation has become an essential strategic choice for enterprises concerned with survival and long-term development [1, 2].

However, the "digital paradox" indicates that enterprises often fail to achieve expected short-term profit growth despite significant investments in digital transformation. This is because digital transformation is not merely a superficial application of digital technology in production or management. It requires businesses to start from their core business needs and deeply integrate data-driven concepts, methods, and mechanisms into their organizational development strategies. The process involves flexible management and continuous iterative optimization of the entire strategic process. The aim is to foster a new mode of value creation [3, 4]. This raises the question: Can digital transformation, which touches upon core business operations and triggers systemic changes, enhance enterprise resilience and ultimately help businesses adapt to turbulent external environments and manage risks effectively?

Research on the relationship between digital transformation and enterprise resilience primarily unfolds from two aspects. The first aspect centers on the core issue of whether digital technology can foster resilience. Based on information processing theory, one viewpoint posits that digital technology is a measure to overcome risks [5]. Studies focus on digital technology’s impact on various entities’ resilience, such as supply chain resilience [6, 7] and platform ecosystem resilience [8]. Other scholars start from the disruptive consequences of digital technology, suggesting that digital transformation leads to disruptive changes and a surge of uncertainties in enterprises [3, 9]. Consequently, these studies explore the uncertain risks of digital transformation outcomes on organizational vulnerability and emphasize the importance of strengthening the assessment of organizational vulnerability and the management of resilience capabilities in transformation decisions [10].

The second aspect focuses on how digital empowerment can respond to environmental changes and disruptive risks to form resilience capabilities. Digital empowerment theory argues that empowered entities, through capabilities and authority granted by digital technology, can overcome crises, respond swiftly to changes during crises, and acquire necessary life and survival skills in crises [11].

Reviewing the progress in these two areas, the discussion on the relationship between digital transformation and enterprise resilience is emerging. However, current research has several shortcomings: First, existing literature mainly examines enterprise resilience through organizations’ recovery from shocks [12, 13], essentially describing it as a "defensive response" and lacking a long-term perspective on stable development and continuous growth to interpret enterprise resilience and explore the impact of digital transformation on this incremental capability. Second, some literature analyses how digitalization affects enterprise resilience or other performance outcomes from perspectives of cost reduction and innovation, yet the mechanisms of how digital transformation affects enterprise resilience are still unclear; the "black box" of mechanisms remains unopened. Third, existing research often overlooks that the relationship between digital transformation and enterprise resilience likely depends further on internal organizational governance mechanisms, with vague discussions on the boundaries of their effects.

Given these gaps, this paper makes several contributions: ***First***, by adopting an economic resilience index synthesis framework and constructing a multi-indicator enterprise resilience system based on microdata from listed companies, it provides a more reasonable and objective alternative to the single-indicator systems prevalent in previous literature, thus beneficially supplementing the micro-level research on enterprise resilience. ***Second***, based on empowerment theory, this paper investigates the impact mechanisms of digital transformation on enterprise resilience from three aspects: agency costs, information transparency, and financing constraints. Additionally, it provides evidence of the differentiated impact of digital transformation on resilience across various enterprises, clarifying the potential limitations in enhancing enterprise resilience through digital transformation and offering more precise and feasible pathways for comprehensive digital development. ***Third***, by incorporating the level of internal corporate governance into the research framework, this paper analyzes its significant role in enhancing enterprise resilience through digital transformation, thereby enriching research related to corporate governance in the digital era.

## 2. Theoretical analysis

### 2.1. Digital transformation and enterprise resilience

In recent years, scholars have mainly focused their research on the consequences of digital transformation by analyzing how companies utilize digital technologies to achieve process optimization, cost reduction, and innovative models [14]. With deeper exploration into digitalization, researchers have begun to emphasize the impact of digitalization on organizational capabilities. Existing studies have shown that digitalization can form new dynamic capabilities and has a significant positive impact on market agility and absorptive capacity [15, 16]. As a special organizational capability, enterprise resilience is the ability of a company to predict, avoid, and respond to internal and external environmental disruptions [17], emphasizing recovery and healthy growth in adversity [18], and is inevitably also affected by the company’s digital transformation.

Existing research indicates that digital empowerment has an important impact on the formation of resilience. Empowerment from corporate digital transformation includes three dimensions: structural empowerment, resource empowerment, and psychological empowerment [11]. Among them, structural empowerment expands the development space of the empowered subjects by changing organizational structures and other objective conditions; resource empowerment helps the empowered subjects acquire, control, and manage resources; psychological empowerment refers to enhancing the mental cognitive abilities of the empowered subjects. In terms of enterprise resilience, this paper believes that the empowerment effect of digital transformation is mainly manifested in structural and resource empowerment.

From the perspective of structural empowerment, digital transformation is essentially a series of innovative processes and results aimed at allowing enterprises to adapt to survival and development in the digital age. It is a process of external-to-internal impact and destruction, followed by internal-to-external adjustment and transformation. In the "new environment," emerging organizational forms like the sharing economy and platform business models, which are the transformed "new species," promote internal information flow, reduce hierarchy, and shorten the decision chain [15]. This structural flexibility enables companies to respond more quickly to market changes and external shocks, thereby enhancing enterprise resilience. Additionally, the application of digital tools such as collaboration platforms and social media tools promotes communication and collaboration between departments, breaking down traditional information barriers. This enhanced collaboration not only improves the efficiency of problem-solving but also helps companies integrate internal resources and respond quickly to threats during crises [19].

From the perspective of resource empowerment, digital transformation shifts the corporate value creation model from a traditional linear state to a new norm dominated by cross-boundary integration and a digital innovation ecosystem. This means that the ways companies cooperate with the outside world and the avenues for resource acquisition will undergo significant changes. Integrating a collaborative system to build mutually beneficial relationships within companies plays an important role in shaping resilience, compensating for the loss of corporate resources in destructive crises, and plays a substitute and synergistic effect on corporate-related production resource elements [20, 21]. Additionally, digital transformation opens up the exploration and accumulation of new resources, laying the resource foundation for shaping enterprise resilience. Resource-based theory suggests that resources that are valuable, rare, irreplaceable, and difficult to imitate can help companies establish unique competitive advantages [22]. Digital transformation reduces communication barriers between organizational departments, increases the speed of information dissemination and technology learning efficiency, and integrates high-quality knowledge capital and human capital into daily operations during the knowledge-sharing process. This not only enables companies to cope with economic challenges but also promotes the enhancement of enterprise resilience through continuous adjustment and learning.

Based on this, the following hypothesis is proposed:

**Hypothesis 1**: Digital transformation can enhance the level of enterprise resilience.

### 2.2. Mechanisms of digital transformation’s impact on enterprise resilience

According to empowerment theory, this article attempts to propose the mechanisms of digital transformation on enterprise resilience. From the perspective of structural empowerment, digital transformation enhances enterprise resilience by reducing agency costs. From the perspective of resource empowerment, on the one hand, digital transformation alleviates information asymmetry by improving the quality of corporate information disclosure; on the other hand, digital transformation eases corporate financing constraints, thus reducing the impacts of imperfect capital markets. Both aspects enhance enterprise resilience through resource empowerment. The following is a detailed analysis of these mechanisms.

#### 2.2.1. Digital transformation, agency costs, and enterprise resilience

The agency problem between managers and shareholders significantly affects enterprise resilience. In corporate governance, ownership and management are often separated, with shareholders entrusting the management to executives. Due to misaligned interests between managers and shareholders, managers may engage in covert compensations such as excessive perquisites or benefits to achieve "hidden payments" [23]. Managers might also expand investment scales to increase their "control benefits," leading to irrational investment behaviors. Such investments are considered unstable decision-making practices, resulting in inefficient resource allocation and reducing the enterprise’s ability to respond flexibly to risk shocks, thereby lowering resilience levels. Research also indicates that when managers do not prioritize maximizing company interests, resource allocation becomes imbalanced. The higher the self-serving incentives, the more likely managers are to possess excessive economic resources, leading to increased enterprise costs [24]. The struggle between shareholders and managers inevitably generates agency costs, reflected in various business operations such as investment, financing, and management, which can significantly impact the enterprise’s operational activities.

As an emerging tool for oversight and governance, digital technology can effectively mitigate agency issues between managers and shareholders and enhance enterprise resilience through governance empowerment. Specifically, digital transformation helps establish a digital governance system based on data mining, analysis, and application, advancing enterprise supervision and management mechanisms toward more scientific and precise modalities. For instance, accounting information systems, management information systems, and decision support systems established through digital transformation can prevent information distortion and automate management decisions [25]. Supported by digital technologies, managers’ investment decisions are increasingly based on quantitative data analysis rather than subjective judgment. This curtails managers’ discretionary power, constrains opportunistic behaviors, and reduces self-interested actions in the investment process, leading to more scientific and rational investment decisions. Consequently, this alleviates agency problems [26], enhancing the enterprise’s resilience.

#### 2.2.2. Digital transformation, information transparency, and enterprise resilience

According to information asymmetry theory, when one party in market transactions possesses more information than the other, it leads to inefficient resource allocation, manifested as moral hazard and other issues. From a game-theoretic perspective, there are two types of market participants: enterprises and investors. Enterprises typically possess all relevant information about themselves and aim to maximize their profits, while investors must rely on publicly available information to make investment decisions, aiming to achieve as high a return as possible. Under conditions of information asymmetry, enterprises may use private information for self-serving behaviors, leaving investors often passively accepting this situation.

Digital transformation helps enhance an enterprise’s ability to disclose information. Technologies like big data and cloud computing have improved the capabilities of data mining, storage, and analysis, providing enterprises with higher quality and more comprehensive data. Blockchain technology ensures information security, accuracy, and reliability, preventing data tampering. Meanwhile, artificial intelligence enables the intelligent collection and real-time transmission of various information during the production and operation processes. The organic integration of "ABCD" technologies (AI, Blockchain, Cloud, and Data Analytics) optimizes the processes of information collection, processing, analysis, and application, further enhancing the enterprise’s capability for information disclosure [27].

With more transparent information disclosure, investors can accurately distinguish between high-quality and low-quality enterprises, gaining a deeper understanding of enterprise products and services and thus more likely to establish trust in enterprises [28]. Enterprises can also deepen their understanding of investor needs and feedback, adjusting and optimizing their business strategies to avoid decisions and behaviors that may lead to risks. This strategic adjustment further enhances the enterprise’s ability to adapt and resist risks. Moreover, in the digital economy era, enterprises are no longer merely in competitive relationships; there is more emphasis on "co-opetition." On the competitive level, enterprises base their operations on obtaining and applying information gradually optimizing their business processes, products, services, and management strategies. Such optimizations make enterprises more adaptable to changes in the market environment, enhancing their survival and development capabilities in market competition. On the cooperative level, information and resource sharing among enterprises disperse risks. Sharing mechanisms can reduce the risks faced by individual enterprises, providing more resources and capabilities to handle uncertainties, thereby enhancing their resilience.

Combining these two factors, the "co-opetition" relationship among enterprises forms a dynamic balance as information transparency increases. This balance aids enterprises in adjusting their strategies to adapt to and cope with the continuously changing market environment, thereby enhancing their resilience levels.

#### 2.2.3. Digital transformation, financing constraints, and enterprise resilience

Financing capability is crucial for enterprises when facing risk shocks. Adequate liquidity allows enterprises to respond swiftly to unforeseen risks, avoiding debt default or cash shortages. This emergency capacity helps maintain the company’s stable operations and reduces the occurrence of extreme events. Moreover, sufficient capital also provides enterprises with competitive salaries, benefits, and development opportunities, helping attract top talent and enabling rapid recovery from crises and overall governance enhancement. However, the capital market development in China is not fully mature, and enterprises face varying financing constraints.

From the perspective of financing constraints, digital transformation can enhance an enterprise’s ability to access resources, thereby alleviating these constraints. On one hand, the digital transformation of enterprises aligns closely with the national "Digital China" strategy. Enterprises that implement digital transformation in accordance with national policies are more likely to gain recognition from stakeholders such as governments and regulatory agencies. This means they can access more resources and enjoy preferential policies, relieving internal financial pressures. In the context of the rapidly developing digital economy, digital transformation has become a societal consensus. Market investors have high positive expectations for enterprises that implement digital transformation, making these enterprises hotspots for capital investment, hence experiencing lower financing constraints. Additionally, according to signaling theory, digital transformation, as an observable and positive action, can send positive signals about an enterprise to the market [29]. Implementing digital transformation indicates an enterprise’s innovation awareness and capability to adapt to market changes.

In traditional bank-firm relationships, banks often face challenges in making lending decisions. However, as digital transformation progresses, enterprises can send positive signals that reduce banks’ information search costs and risk assessment difficulties. This reduces banks’ concerns about credit risk, thus easing the enterprises’ financing constraints. Overall, digital transformation can enhance an enterprise’s ability to access resources and decrease the extent of its financing constraints. Lower financing constraints improve an enterprise’s debt repayment capacity, reducing the likelihood of financial distress, thereby effectively enhancing enterprise resilience levels.

**Fig 1:**
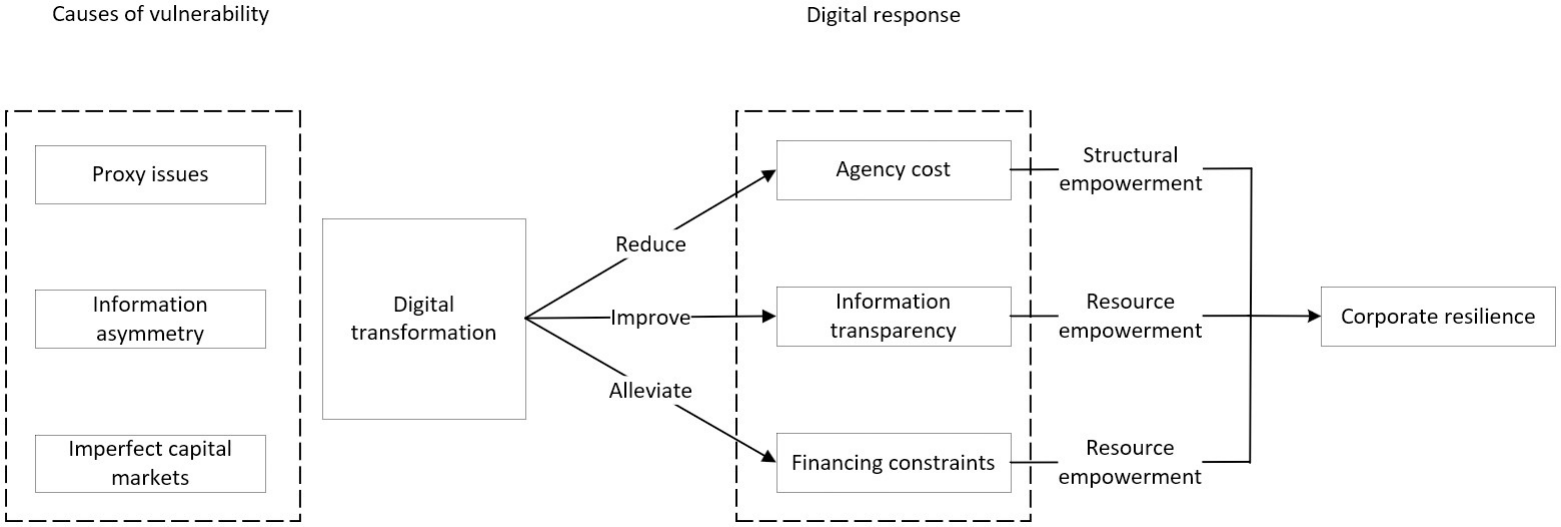
Mechanism framework diagram.

In summary, when companies face risks, issues such as principal-agent problems, information asymmetry, and imperfect capital markets can all contribute to corporate vulnerability. Digital transformation enhances enterprise resilience through structural and resource empowerment. The detailed research framework is shown in Fig 1:

Based on the above discussion, the following hypothesis is proposed:

**Hypothesis 2**: Digital transformation promotes enterprise resilience by reducing agency costs, increasing information transparency, and alleviating financing constraints.

## 3. Research design

### 3.1. Sample selection and data source

This study selects Chinese A-share listed companies from 2007 to 2021 as the research sample. The related financial data are sourced from the *CSMAR* database and *Wind* database. In addition, the data is processed as follows: ***First***, financial industry companies are excluded. ***Second***, companies missing vital financial data are excluded. ***Third***, companies that are ST, *ST, suspended from listing, or delisted are excluded. ***Fourth***, some continuous variables are subjected to natural logarithm transformation and tail trimming at the 1% and 99% levels. A total of 22,784 observations were obtained.

### 3.2. Variable definition

#### 3.2.1. Dependent variable: Enterprise resilience

Resilience refers to the ability of an economy to quickly respond and adapt to external shocks or internal pressures to minimize negative impacts on its financial stability and economic growth. It reflects the system’s capacity to absorb shocks when subjected to external disturbances [30], the ability to return to its original state after experiencing negative impacts [31], and the ability of the system to reach a new state through new growth paths [32]. While scholars have rarely measured resilience at the microeconomic level, this paper posits that enterprise resilience manifests in various aspects of company operations. Using a single indicator to measure enterprise resilience can be specific and heterogeneous and may not accurately reflect the true level of enterprise resilience. Measuring enterprise resilience from multiple dimensions is more scientifically sound and can reflect the resilience of all aspects of enterprise development.

Drawing on the economic resilience analysis frameworks [33, 34], this study attempts to construct micro-level enterprise resilience indicators across three dimensions: defensive resistance capacity, adaptive recovery ability, and transformative learning ability. Detailed secondary indicators, their connotations, and attributes are presented in Table 1. This study first uses the entropy weight method to determine the weights of the secondary indicators and then employs the TOPSIS evaluation method to comprehensively measure the enterprise resilience level. The specific calculation steps are as follows:

**Table 1:** Enterprise resilience indicator system.

First-Level Indicator	Second-Level Indicator	Indicator properties
Resilience to risk	Working capital	Positive
	Equity ratio	Negative
	Tangible net worth debt ratio	Negative
	Equity free cash flow	Positive
Restoring adaptive capacity	Total asset growth rate	Positive
	Net profit	Positive
	Revenue	Positive
Transformative learning capacity	Amount of R&D investment	Positive
	Number of R&D staff	Positive
	Patent citation	Positive

Data Standardization: Standardize the initial data matrix *X* = (*X*_*ij*_)_*m×n*_ to obtain the matrix *R* = (*r*_*ij*_)_*m×n*_. If j is a positive indicator, process according to Eq (1); if j is a negative indicator, process according to Eq (2). To ensure the feasibility of subsequent calculations, let min *r*_*ij*_ = 0.0001.


$$r_{ij}=\frac{X_{ij}-\text{min}x_{j}}{\text{max}x_{j}-\text{min}x_{j}}$$ (1)

$$r_{ij}=\frac{\text{max}x_{ij}-x_{ij}}{\text{max}x_{j}-\text{min}x_{j}}$$ (2)

Calculate the weight of each indicator *P*_*ij*_:

$$P_{ij}=\frac{r_{ij}}{\sum_{i=1}^{m}r_{ij}}$$ (3)

Calculate the entropy value of each indicator, where the entropy value of the jth indicator *e*_*j*_ is:

$$e_{j}=-\frac{1}{\text{ln}\;m}\sum_{i=1}^{m}p_{ij}•\text{ln}\;p_{ij}$$ (4)

Based on the entropy value of each indicator, the weight of the jth indicator is calculated:

$$w_{j}=\frac{\left(1-e_{j}\right)}{\sum_{j=1}^{n}\left(1-e_{j}\right)}$$ (5)

The decision matrix *Z* = (*z*_*ij*_)_*m×n*_ is calculated from the normalized matrix *R* = (*r*_*ij*_)_*m×n*_ with Eq (5):

$$z_{ij}=w_{j}r_{ij}$$ (6)

The positive ideal solutions $z_{j}^{+}$ and negative ideal solutions $z_{j}^{-}$ for each indicator are then determined:

$$z_{j}^{+}=\left\{\begin{array}{l} \text{max}\;\left\{z_{ij}\right\},\text{j}\;\text{is}\;\text{a}\;\text{positive}\;\text{indicator}\\ \text{min}\;\left\{z_{ij}\right\},\text{j}\;\text{is}\;\text{a}\;\text{negative}\;\text{indicator} \end{array}\right.,j=1,\cdots ,n$$ (7)

$$z_{j}^{-}=\left\{\begin{array}{l} \text{min}\;\left\{z_{ij}\right\},\text{j}\;\text{is}\;\text{a}\;\text{positive}\;\text{indicator}\\ \text{max}\;\left\{z_{ij}\right\},\text{j}\;\text{is}\;\text{a}\;\text{negative}\;\text{indicator} \end{array}\right.,j=1,\cdots ,n$$ (8)

Calculate the distance from each indicator to the positive and negative ideal solutions:

$$d_{i}^{+}=\sqrt{\sum_{j=1}^{n}{\left(z_{ij}-z_{j}^{+}\right)}^{2}},i=1,\cdots ,m$$ (9)

$$d_{i}^{-}=\sqrt{\sum_{j=1}^{n}{\left(z_{ij}-z_{j}^{-}\right)}^{2}},i=1,\cdots ,m$$ (10)

Calculate the closeness of the ideal solution for each object *C*_*i*_:

$$C_{i}=\frac{d_{i}^{-}}{d_{i}^{+}+d_{i}^{-}}$$ (11)

Where *C*_*i*_ denotes the closeness of each object to the optimal goal, the larger it is, the stronger the enterprise’s resilience, and vice versa, the weaker the enterprise’s resilience.

#### 3.2.2. Independent variable: Digital transformation

The concept of corporate digital transformation is richly detailed, drawing from existing literature [35], this paper defines corporate digital transformation as the use of new generation digital technologies such as artificial intelligence, big data, cloud computing, and blockchain to digitally overhaul and enhance the existing technological systems and production processes, thereby achieving a high-level transformation characterized by the gradual optimization of production methods and steady improvement in management levels. Enterprises’ application of digital technology is primarily manifested as a series of transformation processes.

Following the approach [35], this study constructs a data pool by utilizing web crawling technology to extract textual information from the annual reports of listed companies to form a data pool. Secondly, it identifies five aspects of corporate digital transformation and 76 characteristic keywords. Finally, the data pool and keywords are matched, categorized, and counted to obtain the final aggregated frequency of words. After normalization, this frequency provides a comprehensive index to measure the extent of corporate digital transformation.

#### 3.2.3. Control variable

A series of control variables were introduced to eliminate potential confounders by drawing on existing literature [36, 37]. The selected control variables include the number of employees in the firm, the firm’s total assets, the asset-liability ratio, the equity concentration, the fixed assets, the turnover ratio of the total assets, and Tobin’s Q value. The descriptive statistics of each variable are shown in Table 2.

**Table 2:** Descriptive statistics.

Variable	N	Mean	Sd	Min	p50	Max
Fr	22784	1.032	3.370	0.064	0.399	96.61
Dt	22784	0.002	1.004	-0.595	-0.342	13.48
Te	22784	7.784	1.202	3.178	7.679	13.22
Asset	22784	22.18	1.297	17.81	22.00	28.64
Lev	22784	0.412	0.208	0.008	0.403	4.995
Tps	22784	0.532	0.154	0.069	0.531	1
Fa	22784	20.31	1.627	11.60	20.18	27.28
Ttc	22784	0.150	0.121	0	0.123	2.319
TobinQ	22784	2.160	1.786	0.012	1.695	92.25

### 3.3. Benchmark model

Based on the analysis in the previous sections, the benchmark regression model set in this article is as shown in Eq (12):

$$Fr_{i,t}=\alpha_{0}+\beta Dt_{i,t}+\varphi Control_{i,t}+\sum Firm+\sum Year+\varepsilon_{i,t}$$ (12)

Here, the dependent variable Fr represents the resilience of the enterprise, the core explanatory variable Dt represents the enterprise’s degree of digital transformation, and Control represents a series of control variables. In addition, the firm represents individual fixed effects used to control factors at the company level that do not change over time. Year means time-fixed effects, used to control exogenous shocks at the macro level. The primary focus is on the significance of the regression coefficient β of digital transformation, which is expected to be positive.

## 4. Empirical result

### 4.1. Benchmark regression

Table 3 reports the baseline regression results for the impact of digital transformation on enterprise resilience. Column (1) of Table 3 shows the OLS regression results, including only the core explanatory variable, where it can be observed that the regression coefficient for digital transformation is significantly positive at the 1% level. Column (2) of Table 3 presents the OLS regression results after including control variables, indicating that digital transformation significantly enhances enterprise resilience at the 5% level. To ensure the reliability of the research results, the paper also conducts panel data analyses with two-way fixed effects and high-dimensional fixed effects regressions, as shown in Columns (3) and (4) of Table 3. It is found that whether controlling for time and individual fixed effects or further controlling for province-year and industry-year interaction fixed effects, the relationship between digital transformation and enterprise resilience is significantly positive, at least at the 5% level. These results suggest that digital transformation improves enterprise resilience, supporting hypothesis 1.

**Table 3:** Benchmark regression results.

	(1)	(2)	(3)	(4)
	Fr	Fr	Fr	Fr
Dt	0.175***	0.059**	0.092**	0.087***
	(5.60)	(2.24)	(2.31)	(4.82)
Te		0.676***	0.163***	0.181***
		(15.74)	(3.51)	(6.21)
Asset		0.944***	0.407***	0.356***
		(16.24)	(10.67)	(11.25)
Lev		-0.881***	-0.258***	-0.203***
		(-6.31)	(-3.27)	(-2.86)
Tps		1.020***	0.317	0.693***
		(6.00)	(1.55)	(5.61)
Fa		-0.240***	0.012	0.007
		(-8.55)	(0.62)	(0.35)
Ttc		0.928***	0.798***	0.799***
		(5.69)	(3.54)	(7.33)
TobinQ		0.110***	0.027***	0.025***
		(8.87)	(4.32)	(3.90)
_cons	1.032***	-20.846***	-9.459***	-8.888***
	(46.29)	(-17.50)	(-10.30)	(-15.98)
Firm FE	NO	NO	YES	YES
Year FE	NO	NO	YES	YES
Industry×Year FE	NO	NO	NO	YES
Province×Year FE	NO	NO	NO	YES
N	22784	22784	22784	22409
R^2^	0.003	0.214	0.154	0.915

This table reports the baseline regression results of the impact of digital transformation on enterprise resilience.

*, * *, and * ** indicate significance at 10%, 5%, and 1% significance levels, respectively. The numbers in the parenthesis correspond to the t-value, the same as below.

### 4.2. Addressing the endogeneity problem

#### 4.2.1. Instrumental variable method

The findings of the benchmark regression suggest that digital transformation can enhance the resilience level of enterprises. However, this conclusion may face endogeneity issues due to reverse causality. On the one hand, digital transformation can improve the resilience of enterprises in coping with market changes and risks. On the other hand, enterprises with high resilience can withstand certain levels of risk impact when facing market competition and external shocks, which is conducive to promoting digital transformation.

This paper adopts three different instrumental variables to address this issue. Firstly, the region’s scientific and technological expenditure is an instrumental digital transformation variable. On the one hand, technological development and research investment are essential supports for the digitalization process. High levels of scientific and technical expenditure may lead to more technological innovations, infrastructure construction, and talent development, all of which are critical factors in promoting digital transformation, fulfilling the assumption of relevance. On the other hand, local scientific and technological expenditures do not directly affect enterprise resilience, fulfilling the premise of exogeneity.

Additionally, since local scientific and technical expenditures are provincial panel data, which do not match with enterprise-level panel data, following the approach [38], the paper multiplies local scientific and technological expenditures with the average digital transformation of other enterprises in the same industry as the enterprise, using the interaction term as the instrumental variable for the digital transformation of the enterprise. The regression results are shown in Table 4, column (1). Secondly, considering that the impact of digital transformation may have a time lag effect, the lagged digital transformation index is used as an instrumental variable. Table 4 column (2) shows the regression results using the lagged level of digital transformation as the instrumental variable. Lastly, following the research [39], a heteroskedasticity-based instrumental variable is constructed. This instrumental variable handles endogeneity and heteroskedasticity issues under specific conditions, especially when the traditional OLS method assumes homoskedasticity. The regression results are shown in Table 4 column (3).

**Table 4:** Instrumental variable regression results.

VARIABLES	(1)	(2)	(3)
	Fr	Fr	Fr
Dt	0.245**	0.117***	0.221***
	(2.06)	(4.91)	(3.12)
Controls	YES	YES	YES
Firm FE	YES	YES	YES
Year FE	YES	YES	YES
Kleibergen—Paap rk LM statistic	484.595***	8822.602***	43.683***
Cragg-Donald Wald F statistic	435.669	1423.436	107.043
	[16.38]	[16.38]	[64.69]
Observations	22576	19588	22784
R^2^	0.024	0.029	0.146

It can be seen that the Kleibergen-Paap rk LM statistics are all significant at the 1% level, rejecting the null hypothesis of the instrumental variables being unidentifiable, indicating the effectiveness of the chosen instrumental variables. The Cragg-Donald Wald F statistics are all higher than the critical value level of 10% bias in Stock-Yogo, suggesting no weak instrumental variable problem exists. The regression coefficients remain significant at the 1% level after addressing endogeneity, verifying the robustness of the benchmark regression results.

#### 4.2.2. Difference-in-differences method

To further validate that the digital transformation, rather than other potential factors, has a stimulating effect on enterprise resilience, a difference-in-differences (DID) model was constructed to mitigate endogeneity issues. Specifically, China’s launch of a big data research plan in 2015 was used as an exogenous policy shock to construct the DID model. Firstly, a digital transformation dummy variable, Treat, was constructed, defining companies with a digital transformation level above the median in 2015 as 1 and those below as 0. Then, a time dummy variable, Post, was constructed, defining the period after 2015 as 1 and before 2015 as 0. The specific model setup is shown in Eq (13), where the interaction term of Treat and Post is denoted as DID.


$$Fr_{i,t}=\beta_{0}+\beta_{1}Treat_{i}\times Post_{t}+\phi \sum {Control}_{i,t}+\mu_{i}+\eta_{t}+\varepsilon_{i,t}$$ (13)

**Fig 2:**
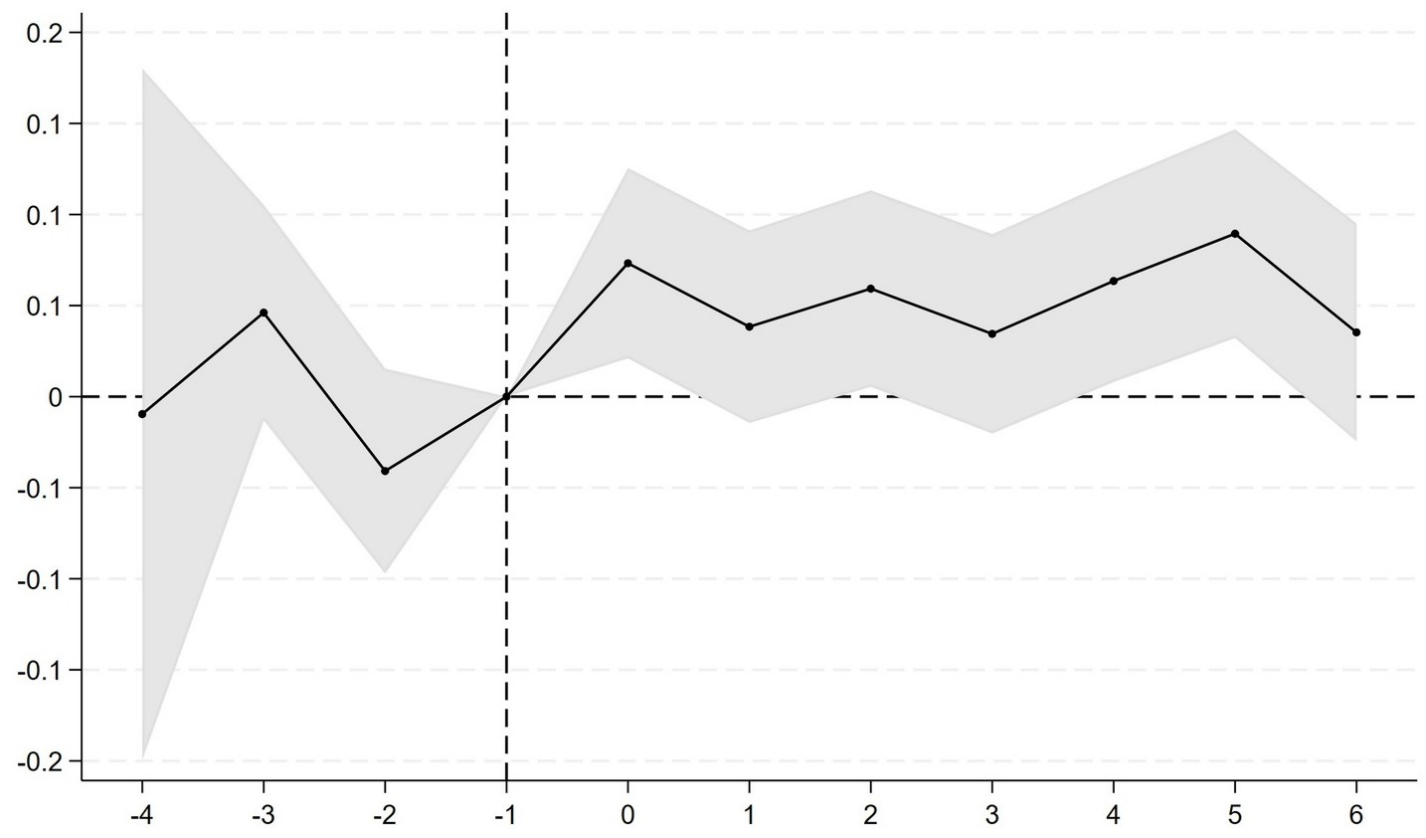
Parallel trend testing.

The basic premise for using DID is that the treatment group and control group companies have parallel trends before the exogenous shock, meaning there is no treatment effect. That is, the trends in the outcome variable are similar between the treatment and control groups. A parallel trend test was conducted to verify this premise, and the results are shown in Fig 2. The figure indicates that there was no significant difference in enterprise resilience levels between the treatment and control groups before the policy, suggesting that the parallel trend assumption is satisfied, supporting the validity of the DID method used in this study.

Table 5, column (1) shows regression results without control variables, and column (2) shows results with control variables, with the DID coefficients significantly positive at the 1% confidence level. Furthermore, to overcome endogeneity issues due to inherent characteristics differences between companies at different levels of digital transformation, the paper used the PSM method for 1:1 nearest neighbor matching of some company characteristic indicators. After passing the stationarity test, the regression was conducted with the PSM-matched sample, and the results, as shown in Table 5 column (3), indicate that the coefficient for corporate digital transformation is significantly positive at the 1% level. These results demonstrate that compared to companies that have not undergone digital transformation, those that have shown a significant improvement in resilience, further verify the baseline regression results.

**Table 5:** DID regression results.

VARIABLES	(1)	(2)	(3)
	Fr	Fr	Fr
DID	0.216***	0.136***	0.137***
	(4.70)	(3.33)	(3.35)
_cons	0.461	-9.478***	-9.551***
	(1.39)	(-11.75)	(-11.77)
Controls	NO	YES	YES
Firm FE	YES	YES	YES
Year FE	YES	YES	YES
N	18698	18698	18693
R^2^	0.181	0.238	0.238

**Fig 3:**
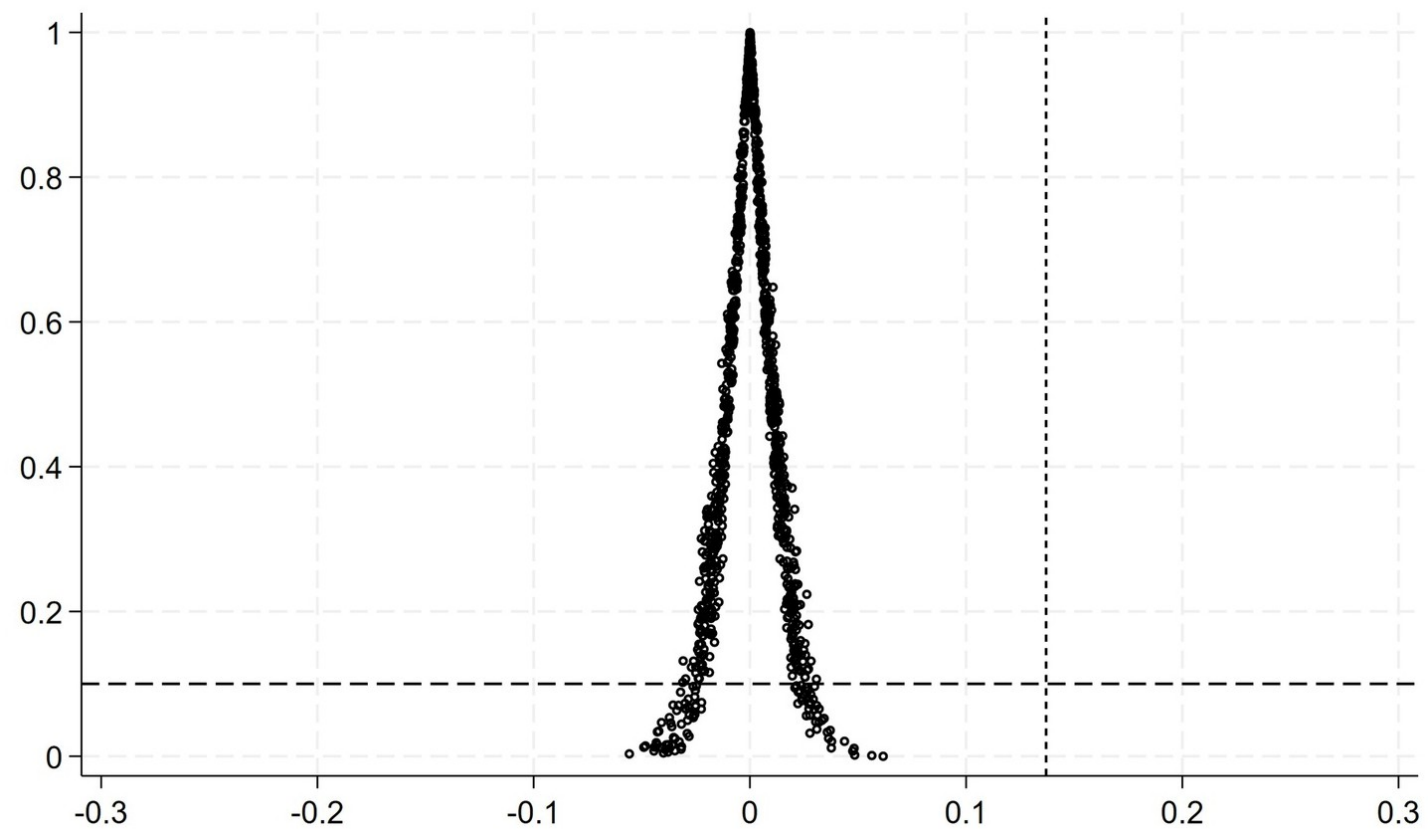
Placebo testing.

Moreover, the paper conducted a placebo control test to reduce the interference of inherent characteristic differences between the treatment and control group companies and simultaneous events. A dummy experimental group was constructed in both company and time dimensions for the placebo test. Based on the regression model of Eq (13), 1000 random sampling simulation regressions were conducted, and the results are shown in Fig 3. The estimated coefficient distribution from the random sampling regression approximates a normal distribution, with a mean close to 0, and no sampling results located to the left of the baseline model coefficient (far less than the DID coefficient of 0.137), indicating that the estimation results of the previous text are credible.

### 4.3. Robustness check

To ensure the reliability of the research conclusions, this article also conducted the following robustness tests.

#### 4.3.1. Replacement of regression model

Considering that the current resilience level of an enterprise may be influenced by the previous period, a dynamic panel model was used to re-evaluate this causal relationship. The regression results are shown in Table 6, column (1). After considering the lagged effect of enterprise resilience, the impact of digital transformation on resilience is significantly positive at the 10% level, consistent with the benchmark regression results. Furthermore, AR(1) is less than 0.1, AR(2) is between 0.1 and 0.25, and the Hansen P-value is insignificant, satisfying the relevant assumptions of SYS-GMM.

**Table 6:** Robustness tests.

VARIABLES	(1)	(2)	(3)	(4)	(5)
	Fr	Fr	Fr	Fr	Fr
L.Score	0.454***				
	(3.19)				
Dt	0.062*	0.014**		0.130***	
	(1.85)	(1.97)		(3.05)	
L.Dt			0.066**		
			(1.97)		
Dtr					0.956**
					(2.04)
Controls	YES	YES	YES	YES	YES
Firm FE	YES	YES	YES	YES	YES
Year FE	YES	YES	YES	YES	YES
_cons	-1.256*	45.695***	-6.830***	0.021	-0.069***
	(-1.74)	(141.37)	(-8.12)	(0.21)	(-46.47)
N	19606	22784	19606	21126	20438
R^2^		0.001	0.131	0.148	0.479

#### 4.3.2. Replacement of dependent variable

The enterprise resilience index was recalculated using the CRITIC weighting method. The CRITIC weighting method, an objective weighting method superior to the entropy weighting method, considers the contrast intensity of the evaluation indicators and the conflict between them to determine their accurate weights. While considering the variability of indicators, this method also accounts for the correlation between them, facilitating a comprehensive and scientific evaluation. Regression results are shown in Table 6, column (2), where the digital transformation coefficient is positive at the 5% confidence level.

#### 4.3.3. Explanatory variables lagged one period

Considering that the impact of enterprise digital transformation might also have a time lag effect, the core explanatory variable lagged by one period and regressed again. As shown in Table 6 column (3), the results reveal that the coefficient estimates are significantly positive at the 5% level.

#### 4.3.4. Excluding special sample

Due to the high level of digitization inherent in high-tech companies, to investigate the causal effects within other companies in the sample, high-tech companies were excluded from robustness testing. The regression results are shown in column (4) of Table 6, where the regression coefficient for digital transformation is positive at the 1% confidence level.

#### 4.3.5. Replacement of independent variable

Existing literature using text analysis to capture the frequency of terms related to digitalization reflects more on the degree of corporate attention to digitalization rather than the actual level of corporate digital transformation, potentially resulting in an embellishment of reality. Drawing on the research [40], this study re-estimates the baseline regression using the ratio of digitization-related intangible assets to total assets in the financial statements of listed companies as a proxy variable for corporate digital transformation. The regression results are shown in column (5) of Table 6.

## 5. Mechanism analysis and heterogeneity test

### 5.1. Mechanism analysis

In the traditional three-step method of mediation effect models, there may be a collinearity issue between the explanatory variables and the mediator variables, which could increase the estimation standard errors and reduce the statistical significance [41]. More importantly, since mediator variables cannot be strictly controlled, the estimation might be biased due to omitting variables that simultaneously affect both the mediator and the explained variables. Additionally, using Sobel tests in the three-step regression analysis framework requires data to follow a normal distribution. Therefore, it is recommended that only the impact of the explanatory variables on the mechanism variables be tested, and the influence of mechanism variables on the explained variables should be discussed theoretically. In light of this, this study directly tests the impact of digital transformation on the mechanism channels and uses Bootstrap for auxiliary judgment. The specific model setup is shown in Eq (14):

$$M_{i,t}=\alpha +\beta X_{i,t}+\delta Control_{i,t}+\mu_{i}+\lambda_{t}+\varepsilon_{i,t}$$ (14)

The subscripts i and t represent the enterprise and the year, respectively. X represents the degree of enterprise digital transformation, M is the mechanism variable, and Control represents a series of control variables related to enterprise resilience. α is the intercept, μ and γ represent individual and time effects, and ε is the random disturbance term. This paper selects three potential mechanism variables for detailed analysis: agency costs, information transparency, and the degree of financing constraints.

First, the management expense ratio (Mfee) is used to measure the agency costs of the company [42], where a higher value indicates higher agency costs. As shown in column (1) of Table 7, the regression results indicate that digital transformation significantly reduces agency costs at the 1% level. Second, information transparency is examined as a mechanism channel, using the KV index to measure the transparency of corporate information [43], with a lower index indicating more transparent information disclosure. The regression results in column (2) of Table 7 demonstrate that digital transformation significantly enhances corporate information transparency at the 1% level. Lastly, the Ww index is used to measure the extent of financing constraints faced by the company [44], where a lower negative value indicates less severe financing constraints. The regression results in column (3) of Table 7 reveal that digital transformation significantly alleviates financing constraints at the 1% level. Additionally, the Bootstrap self-iterative 1000 times indirect effect confidence intervals for all three mechanism channels do not include zero, confirming the robustness of the results. Therefore, research hypothesis 2 is validated.

**Table 7:** Mechanism test results.

VARIABLES	(1)	(2)	(3)
	Mfee	Kv	Ww
Dt	-0.007***	-0.013***	-0.005***
	(-5.62)	(-4.09)	(-2.94)
_cons	0.076***	0.116***	-1.010***
	(48.94)	(839.15)	(-126.86)
Controls	YES	YES	YES
Firm FE	YES	YES	YES
Year FE	YES	YES	YES
Confidence intervals for indirect effects	(-0.006,-0.002)	(0.006,0.014)	(0.004,0.029)
N	22298	21630	19273
R^2^	0.235	0.127	0.024

### 5.2. Heterogeneity test

#### 5.2.1. Heterogeneity test based on internal micro characteristics of enterprise

The sample companies are categorized into labor-intensive, technology-intensive, and capital-intensive types [45]. The regression results displayed in Table 8 columns (1), (2), and (3) show that coefficients are significant at the 1% level for labor-intensive and capital-intensive companies, but the coefficient for technology-intensive companies trends toward zero and is not significant. Specifically, labor-intensive companies can significantly enhance their resilience by adopting technologies like automation and artificial intelligence, which help reduce labor costs, improve production efficiency, and minimize quality variability. For capital-intensive companies, which usually have high market barriers such as economies of scale, technology, and patents, their profit models and business processes are relatively mature and stable. These companies often face significant transformation resistance during digital transformation, hence the relatively smaller impact of digital transformation on resilience than labor-intensive companies. In contrast, technology-intensive companies, which continuously need to try new technologies and business models to cope with intense market competition, often have a higher risk preference, making the positive effects of digital transformation on resilience less apparent in these firms.

**Table 8:** Heterogeneity test based on internal micro characteristics of enterprises.

VARIABLES	(1)	(2)	(3)	(4)	(5)	(6)
	Labor-intensive	Capital-intensive	Technology-intensive	Growth	Maturity	Decline
Dt	0.187***	0.082***	0.007	0.096	0.123**	0.134***
	(2.64)	(3.54)	(0.14)	(1.16)	(2.17)	(2.77)
_cons	-0.021	0.167	0.247	-1.388	0.141	-0.321
	(-0.19)	(0.91)	(0.81)	(-1.21)	(0.29)	(-0.58)
Controls	YES	YES	YES	YES	YES	YES
Firm FE	YES	YES	YES	YES	YES	YES
Year FE	YES	YES	YES	YES	YES	YES
N	19180	1164	2440	3790	8159	9836
R^2^	0.165	0.321	0.117	0.177	0.109	0.105

On the other hand, the sample companies are categorized into growth, maturity, and decline phases for group regression analysis [46]. Empirical results, as shown in columns (4), (5), and (6) of Table 8, indicate that the coefficient for companies in the growth phase is smallest and not significant, the coefficient for mature phase companies is 0.123 and significant at the 5% level, while the coefficient for declining phase companies is the largest and significant at the 1% level. Specifically, companies in the growth phase face high market uncertainty and competitive pressure, focusing more on market share expansion and product innovation, which may lead to a lack of focus on resilience. Companies in the maturity phase have established stable market positions and customer bases, allowing them to focus more on internal management and efficiency improvements. In this stage, digital transformation significantly enhances enterprise resilience.

In contrast, companies in the decline phase face shrinking market shares and declining competitiveness, making digital transformation a critical means of enhancing survival capabilities and competitiveness. In this stage, companies may increase investment in digital transformation to seek breakthroughs and transformation. Overall, the impact of digital transformation on enterprise resilience varies depending on the different life cycle stages of the companies.

#### 5.2.2. Heterogeneity test based on the external macro environment of enterprise

In the research, the Herfindahl-Hirschman Index (HHI) is employed to assess the level of competition within industries, and the sample is divided into monopolistic and competitive sectors [47]. The results, shown in columns (1) and (2) of Table 9, indicate that for firms in monopolistic industries, the coefficient is not significant, whereas for those in competitive industries, the coefficient is 0.139 and significant at the 1% level. This demonstrates that, compared to monopolistic sectors, the positive impact of digital transformation on enterprise resilience is more evident in competitive industries.

**Table 9:** Heterogeneity test based on the external macro environment of enterprise.

	(1)	(2)	(3)	(4)
	Regulated industries	Competitive industry	Low intellectual property protection	High intellectual property protection
Dt	0.102	0.139***	0.041	0.128**
	(0.73)	(3.17)	(1.52)	(2.24)
_cons	-22.732***	-2.190	-9.155***	-10.583**
	(-4.39)	(-1.16)	(-5.76)	(-2.18)
Controls	YES	YES	YES	YES
Firm FE	YES	YES	YES	YES
Year FE	YES	YES	YES	YES
N	4435	18349	13525	9259
R^2^	0.170	0.178	0.236	0.137

More specifically, as an external environmental factor, the level of competition in an industry influences the market pressures firms face, thereby acting as an external governance mechanism. On one hand, industries with higher competition typically emphasize efficiency and innovation. In such industries, firms can better utilize data and information technology through digital transformation to optimize operations, reduce costs, and improve resilience. Conversely, monopolistic industries may have their efficiency and innovation constrained by industry characteristics, limiting firms’ autonomy and innovative capabilities during digital transformation, and thus, the strengthening effect on resilience is relatively weaker. On the other hand, firms in competitive industries must closely monitor market changes. Digital transformation enables these firms to respond more swiftly to these changes. In monopolistic industries, where the market may be more stable, the impact of digital transformation on enhancing resilience might not be as significant as in competitive sectors.

Additionally, intellectual property (IP) protection levels across regions are characterized by averaging the ratios of IP infringement cases filed per total population and the number of lawyers per total population. Regions above the median level of IP protection are considered to have high IP protection, while those below have low protection. Empirical results, shown in columns (3) and (4) of Table 9, indicate that the coefficient for firms in high IP protection areas is 0.128 and significant at the 5% level, whereas the coefficient for firms in low IP protection areas is insignificant.

In detail, when IP protection is robust, firms are more likely to benefit from their innovative outputs and technological advantages, which helps them maintain a competitive edge during digital transformation. Furthermore, firms in environments with high IP protection are more likely to obtain external investment and financing support, as investors and financial institutions are generally more inclined to invest in firms with IP protection advantages. Thus, regional IP protection regimes can influence the resilience-enhancing effects of digital transformation on businesses.

## 6. Further analysis

To further explore the impact of digital transformation on enterprise resilience, the study first uses a static panel threshold model for testing. The main idea is to find a threshold value within the model that leads to significant structural changes on both sides of the threshold, thus avoiding subjective bias in the model setting. Since resilience has temporal continuity, the static panel threshold model cannot include the lagged value of the dependent variable and also faces endogeneity issues. Therefore, a dynamic panel model is employed to test this issue, with the specific model setting shown in Eq (15).

$$Fr_{i,t}=\alpha_{0}+\alpha_{1}Fr_{i,t}+\beta_{1}Dt_{i,t}\times I(•)(Cgl_{i,t}\leq \gamma )+\beta_{2}Dt_{i,t}\times I(•)(Cgl_{i,t}>\gamma )+\phi X_{i,t}+\varepsilon_{i,t}$$ (15)

Where *i* = 1,2,⋯,*N* represents individuals, and *t* = 1,2,⋯,*T* represents time, enterprise resilience and digital transformation are the dependent and explanatory variables, respectively. The level of corporate governance is constructed and set as the threshold variable [48], with lower values indicating higher levels of corporate governance. *I*(•) is an indicator function that takes the value of 1 when the corresponding condition is satisfied and 0 otherwise. This article sets the Bootstrap iteration number to 1000 times. First, the threshold effect of the static panel threshold model is tested. As shown in Table 10, the corporate governance level, the threshold variable, passes the single threshold test at the 5% level, and the double threshold test P-value is insignificant, suggesting that the model has only one threshold value. Additionally, since the dynamic panel threshold model estimation requires balanced panel data, the study processes the unbalanced data into balanced data, resulting in a sample size of 11274. Following the selection of instrumental variables in the previous text, the regional science and technology expenditure is introduced into the dynamic panel threshold model to address endogeneity issues.

**Table 10:** Threshold effect test.

Threshold variables	Threshold number	Symbolic	Threshold value	P-value
Cgl	Single-threshold	R1	-0.9273	0.020
	Double-threshold	R2	-1.6722	0.270

After obtaining the specific threshold value, panel threshold regression is conducted by selecting appropriate control variables. The results are shown in Table 11, where the explanatory variable is digital transformation, and the dependent variable is enterprise resilience. Column (1) presents the estimation results of the static threshold model, while columns (2) and (3) show the estimation results of the dynamic threshold model. According to the regression results in column (3), the impact of digital transformation on enterprise resilience exists in two regimes: when the level of corporate governance is high (Cgl < -0.649), digital transformation has a significant enhancing effect on enterprise resilience, with an impact coefficient of 0.407, significant at the 1% level; when the level of corporate governance is low (Cgl > -0.649), digital transformation still significantly enhances enterprise resilience, but the impact coefficient is reduced to 0.124, significant at the 10% level.

**Table 11:** Threshold regression results.

	(1)	(2)	(3)
	Static Thresholds	Dynamic thresholds	Dynamic thresholds
Threshold value	-0.9273**	-0.649**	-0.649***
Dt(Cgl<r)	0.405**	1.114***	0.407***
	(2.48)	(5.09)	(2.56)
Dt(Cgl>r)	0.103***	0.468***	0.124*
	(3.70)	(-2.87)	(-1.92)
_cons	1.438	1.377***	13.239**
	(0.79)	(2.92)	(2.43)
Controls	YES	NO	YES
N	21512	11274	11274

Specifically, digital transformation, as a manifestation of the enterprise’s technological level, and corporate governance, as a reflection of the enterprise’s management level, affect enterprise resilience together. When the level of corporate governance is high, the promoting effect of digital transformation on enterprise resilience may be strengthened. Firstly, effective corporate governance provides a more precise decision-making framework, ensuring fairness, compliance, and transparency in the decision-making process. This will benefit enterprises in more effectively addressing uncertainties during digital transformation; secondly, digital transformation may also trigger a series of technological, human resource, and market risks. Under good corporate governance conditions, enterprises can establish a more comprehensive and in-depth risk assessment and management system, thus better controlling risks during digital transformation; finally, digital transformation is not the sole factor affecting enterprise resilience. The rational allocation of resources such as funds, talent, technology, and information is crucial for enhancing resilience, and a good level of corporate governance can promote optimal resource allocation within enterprises.

In summary, corporate governance and digital transformation have a significant synergistic effect on enhancing enterprise resilience. They are not isolated but represent a complex dynamic interaction process. A deeper understanding of this is of great theoretical value and has significant implications for practical enterprise management and policy formulation. Enterprises face increasingly complex external environments, especially in the current era of accelerating digital transformation. In this context, corporate governance becomes particularly important. It is an intrinsic driving force within the enterprise, guiding and supporting digital transformation and enhancing enterprise resilience.

## 7. Conclusion and implication

### 7.1. Research conclusion

Based on annual data of A-share listed companies from 2007 to 2021, this paper constructs a resilience index using the entropy weight TOPSIS method and conducts a series of empirical tests on the impact of digital transformation on enterprise resilience. The findings are as follows: ***First***, digital transformation significantly enhances the level of enterprise resilience, and this conclusion remains valid after a series of robustness tests. ***Second***, digital transformation promotes enterprise resilience by reducing agency costs, enhancing information transparency, and alleviating financing constraints. ***Third***, heterogeneity tests indicate that the effect of digital transformation on enhancing resilience is more significant for labor and capital-intensive companies and those in the maturity and decline phases; for companies in competitive industries and regions with high intellectual property protection, the effect is relatively greater. ***Fourth***, the positive effect of digital transformation on enterprise resilience is influenced by the level of corporate governance, with higher levels of corporate governance enhancing the effect of digital transformation on resilience.

### 7.2. Research implication

The conclusions of this paper reveal the relationship between digital transformation and enterprise resilience to some extent, and based on the findings, the following policy suggestions are proposed:

***First***, companies should deeply understand the importance of digital transformation to their resilience. Historical trends show that digitization is not only a result of the informatization process but also symbolizes the deep evolution of informatization and is the core foundation of building intelligent enterprises. Companies should actively implement and comprehensively apply digital technologies such as the internet and big data, fully exploiting the potent potential of digital technology to enhance their resilience.

***Second***, governments should introduce policy measures to create a favorable external environment for digital transformation. Initially, governments can establish related information disclosure requirements and guidelines, encourage companies to adopt advanced information technologies, and strengthen cooperation with third-party information disclosure service agencies to enhance the accuracy and timeliness of information. This ensures companies fully disclose essential financial and operational information during digital transformation; next, governments can promote financial institutions to provide preferential loans to companies undergoing digital transformation or provide fiscal subsidies and tax incentives to eligible companies, supporting their financing through various channels and enhancing financing efficiency; finally, governments can enact related regulations, requiring companies to improve internal governance systems and strengthen internal audits, risk assessments, and other regulatory aspects to ensure effective risk management during digital transformation.

***Third***, the differential performance of digital transformation in various companies should be fully considered. Companies should develop feasible digital transformation strategies according to their characteristics and industry environment, fully leveraging the promotional effect of digital transformation on resilience; governments should actively create a favorable external business environment, enhance the protection of intellectual property rights for digital technologies and data assets, relax regulations orderly, and promote free market competition.

***Fourth***, digital transformation is not the only factor affecting enterprise resilience. Companies must judge the situation during the digital transformation process and find the optimal balance between digital transformation and other key operations to achieve optimal resource allocation.

### 7.3. Research limitation and prospect

The main limitations of this paper include the following three points. ***First***, in terms of variable measurement, future research could explore enterprise resilience from different perspectives, such as the long-term survival rate of companies. ***Second***, in analyzing mechanisms, this paper introduces agency costs, information disclosure, and financing constraints as mediating paths to explore how digital transformation affects enterprise resilience. Future research is needed to unravel further the "black box" of the mechanisms of digital transformation and its economic benefits. ***Third***, in terms of research subjects, future studies could consider the ecosystem in which a company operates, examining whether the positive effects of digitization on a company can spill over to the supply chain industry chain, thereby enhancing the overall resilience and risk resistance of the supply chain industry chain.

## Supporting information

S1 File (TXT)

S2 File (XLSX)

S3 File (TXT)

S4 File (XLSX)

S5 File (XLSX)
